# Influence of Interocular Differences and Alcohol Consumption on Binocular Visual Performance

**DOI:** 10.3390/ijerph20031751

**Published:** 2023-01-18

**Authors:** Francesco Martino, José J. Castro-Torres, Miriam Casares-López, Sonia Ortiz-Peregrina, Pilar Granados-Delgado, José R. Jiménez

**Affiliations:** Laboratory of Vision Sciences and Applications, Department of Optics, Faculty of Sciences, University of Granada, Avenida Fuentenueva s/n, 18071 Granada, Spain

**Keywords:** alcohol consumption, retinal–image quality, straylight, interocular differences, binocular summation, visual performance, induced forward scattering

## Abstract

The purpose of this study was to assess the influence of a moderate breath–alcohol content (BrAC of 0.40 mg/L) on binocular visual performance for different visual functions after inducing different levels of interocular differences with the use of filters. A total of 26 healthy young subjects were enrolled. The participants participated in two sessions: one without alcohol consumption and another after alcohol consumption. In each session and for the different filter conditions (subjects were wearing Bangerter foil of 0.8 and BPM2 fog filter on the dominant eye), monocular and binocular visual function was evaluated by measuring visual acuity, contrast sensitivity, visual discrimination capacity (and successively by calculating their corresponding binocular summations) and stereopsis (near and distance stereoacuity). In addition, interocular differences were calculated for different retinal–image quality and straylight parameters. All monocular and binocular visual functions were analyzed and stereopsis was significantly impaired by alcohol and filters (*p* < 0.05). Interocular differences for different ocular parameters and binocular summations for visual parameters were negatively affected by filters but not alcohol. Significant correlations (averaging all the experimental conditions analyzed) were found, highlighting: the higher the interocular differences, the lower the binocular summation and the poorer the stereopsis and, therefore, the worse the binocular visual performance.

## 1. Introduction

Alcohol is the most widely consumed psychoactive substance in the world, which causes a severe impact on the health of the world’s population and is responsible for more than three million deaths around the world [1]. With a prevailing psychoactive effect, alcohol acts and interacts negatively on and with the central nervous system, which reduces the speed of neuronal processing and decreases neural transmission [2]. In these circumstances and depending on the quantity of alcohol ingested, overall visual function is prejudiced by this substance. Specifically, alcohol consumption negatively affects several monocular and binocular visual parameters, such as visual acuity [3,4], contrast sensitivity [5,6,7,8], and visual discrimination capacity at night (through the perception of halos) [9,10], thus, deteriorating visual performance. Indeed, the negative impact of alcohol consumption on our natural vision condition, i.e., on binocular vision, has been demonstrated in several studies [11,12,13]. In this condition, a worsening in normal binocular vision perturbs important daily visual tasks, such as driving [5,11,14,15], reading [16,17], fine motor skills [18,19] or manual dexterity [16,20]. To assess binocular visual performance, binocular summation on different visual functions [21,22,23,24,25,26,27] and stereopsis [28,29,30,31,32] are widely used. Binocular summation is a ratio quantifying the superiority (or not) of the binocular visual system compared to the monocular one for a specific visual function. Stereopsis is the visual system’s capacity and quality to see the surrounding environment in-depth. In addition to alcohol intake, binocular visual performance can be affected by several factors, such as defocus and pupil size [33]. One of these factors is the interocular differences (differences between the two eyes concerning a determined ocular parameter) which negatively disturb binocular vision. Increased interocular differences lead to that deterioration through decreased binocular summation [26,27,34,35,36,37] and the impairment in stereopsis [27,38,39,40,41,42]. For instance, in clinical applications, Jimenez et al. [43] referred that increased interocular differences in corneal asphericity induced by refractive surgery, such as LASIK (Laser-Assisted in Situ Keratomileusis), impaired binocular visual performance (stereopsis). Interocular differences can also be willingly induced using emmetropization techniques, such as monovision to correct presbyopia (the dominant eye is corrected for distance viewing and the non-dominant eye for near viewing) [44,45,46]. On the other hand, an important phenomenon that can increase interocular differences is intraocular scattering, which can cause a veiling luminance on the retina resulting in what is known as retinal straylight. Indeed, if intraocular scattering occurs in one eye more than in the other, it can cause a greater interocular difference affecting binocular visual performance [27,34,47]. The increment of intraocular scattering causes a deterioration of retinal image quality [29] and visual disturbances, such as disability glare (decreasing contrast sensitivity) [48,49,50] or night vision disturbances (such as halos) [51,52,53]. These visual disturbances can also be associated with different ocular pathologies, such as keratitis [54,55], age-related macular degeneration (AMD) [56,57], cataract [58,59], and amblyopia [60,61]. Considering ocular pathologies, some filters have been used both to simulate an early cataract, such as the BPM2 fog filter [62,63], and for the treatment of amblyopia, such as Bangerter foils [64,65]. In addition, these filters have also been used to simulate different degrees of interocular difference to better understand the effect on binocular visual performance [27]. However, to our acknowledge, no previous studies have investigated the effect of interocular differences on binocular visual performance under the influence of alcohol consumption. To improve our understanding of this field, it could be of interest to study how different degrees of interocular differences induced by filters influence binocular visual performance with and without alcohol consumption to better understand visual health risks.

Taking into consideration the aforementioned studies and arguments, our hypotheses are, firstly, that binocular visual performance (evaluated with binocular summation for several visual functions and stereopsis) could deteriorate with the increase of induced interocular differences in some visual parameters (assessing intraocular scattering, retinal-image quality and straylight) and secondly, that the worsening of binocular vision could be intensified by alcohol intake. Regarding this aspect, the effects of moderate alcohol consumption are relevant since real-life social drinking is a common and accepted practice in a wide variety of cultures and countries. Moreover, a moderate blood alcohol level of 0.08% (equivalent to a breath alcohol content of 0.40 mg/L) is of special interest, as it is the highest most-common legal limit for driving around the world [1] and has been shown to affect some aspects of vision negatively [11].

Therefore, the aim of this study was to assess the influence of a moderate breath alcohol content (BrAC) of 0.40 mg/L on binocular visual performance through the quantification of binocular summation for different visual functions, such as visual acuity, contrast sensitivity function, and visual discrimination capacity and stereopsis (at a distance and near) after inducing different levels of interocular difference with the use of filters.

## 2. Materials and Methods

### 2.1. Patients

A total of 26 young, healthy subjects (12 females and 14 males) with a mean age of 26.0 ± 4.6 years and a mean body mass index (BMI) of 22.6 ± 3.0 kg/m^2^ were enrolled in this crossover study. The mean refractive error (spherical equivalent) was −1.16 ± 1.77 D. The inclusion criteria were: best-corrected monocular and binocular visual acuity ≥1.0 (in decimal notation), normal stereoacuity near and at a distance (40 arcsecs or lower), no pathology or pharmacological treatment that could affect visual performance, and no contraindication for alcohol consumption—being a social drinker with a score eight or less obtained on the alcohol use disorders identification test (AUDIT) [66,67].

Finally, sensory ocular dominance was determined using the line anterior to the best visual acuity and alternating a +1.50 D lens in front of each eye under binocular viewing conditions. The dominant sensory eye was the eye with the positive lens reporting the most blurred vision [68]. Before beginning the experiment, all participants signed an informed consent form in accordance with the Declaration of Helsinki. The study was approved by the Human Research Ethics Committee of the University of Granada.

### 2.2. Filters

Two types of filters were assessed in the present study. Firstly, a Bangerter foil (Ryser Optik, St Gallen, Switzerland) corresponding to a grade 0.8 (BF_0.8) was used to degrade the retinal image quality. The 0.8 value corresponds to the theoretical visual acuity in decimal notation obtained when viewing through the foil (assuming an initial visual acuity of 1.0 or better). The BF_0.8 foil used is formed by microbubbles which produce image distortions affecting visual acuity and contrast sensitivity [27,69]. As it is well established, Bangerter foils are prescribed for the treatment of amblyopia, mainly in children [61,70]. Secondly, the Black Pro-Mist 2 fog filter (BPM2, Tiffen, Hauppauge, NY, USA) was used because it has been proven to be suitable for the simulation of an early cataract [62,69]. This type of filter is commonly used in film and photography. It shows a structure characterized by grain, which is variable in size and irregular in shape, as shown in macro-photographs of this filter [27,69]. In the study, the two filters: BF_0.8 and BMP2, were previously placed onto an artificial eye (TOPCON, spherical refraction −5.5 D) and tested using the OQAS II (Optical Quality Analysis System II, Visiometrics, Spain) to objectively assess the optical quality of the artificial eye wearing each of the two filters. As a result, this procedure allowed us to avoid inter-individual variability and to confirm the human observers’ measurements [27,69]. In practice, each of the two filters was placed on the subject’s dominant eye and on both eyes in the case of the Bangerter foil. The BPM2 filter was mounted into Knobloch K-2 shooting glasses (Knobloch Optik GmbH. Karlsruhe, Germany) using a filter adapter, allowing us to fix the lens holder with the BPM2 filter centered in front of the eye. The BF_0.8 foil was placed on ophthalmic lenses, with no optical power, mounted in identical optical frames (for each eye, a frame for the BF_0.8 affecting one eye).

### 2.3. Procedures and Alcohol Consumption

The participants participated in two sessions: one under natural conditions (baseline, without alcohol consumption) and another after alcohol consumption (aAC). In each session and for the different filter conditions, visual function was measured monocularly, and binocularly and ocular parameters were measured monocularly. All the visual tests were randomized in the two experimental sessions (baseline and aAC) to avoid learning effects. None of the sessions took more than 90 min in order to avoid visual fatigue. Finally, both sessions were performed with an interval of one week between them to limit the order effect. In the session with alcohol intake, the participants consumed a mixed alcohol beverage (67% orange juice and 33% vodka). We measured the breath alcohol content (BrAC), defined in milligrams of ethanol per liter of exhaled air (mg/L), using the Alcotest 6810 breath analyzer (Dräger Safety AG& Co. Lubeck, Germany) which provides a good reproducibility [71]. The BrAC required was 0.40 mg/L corresponding to a moderate alcohol content. To calculate the quantity of alcohol ingested for each participant, we used an improved version of the Widmark formula [72]. Each participant consumed the corresponding dose of alcohol within a time of 30–40 min. During the experimental session with alcohol consumption, the BrAC was measured every 20 min to check that it was stabilized at 0.40 mg/L. The mean BrAC obtained for all participants was 0.40 ± 0.04 mg/L. The participants were informed that they were consuming alcohol in order to test them in a common real-world situation.

### 2.4. Visual Function

#### 2.4.1. Visual Acuity and Contrast Sensitivity

In this study, visual acuity and contrast sensitivity were measured using the Pola VistaVision monitor (DMD MedTech, Villarbasse, Torino, Italy). Visual acuity was quantified monocularly (for both eyes) and binocularly in decimal notation under photopic lighting conditions at a distance of 5.5 m. The evaluation of the contrast sensitivity was performed monocularly and binocularly under mesopic lighting conditions at a distance of 3 m. The luminance background was 60 cd/m^2^. To this end, the participants had to indicate different decreasing contrasts on the side the grids were inclined (right, left, or vertical) until they could not recognize the grid. Eight spatial frequencies were evaluated: 0.75, 1.5, 3, 6, 12, and 18 cycles per degree (cpd). In order to report a single value characterizing contrast sensitivity for each condition, we averaged contrast sensitivity for these spatial frequencies, as has been performed in other studies [5,27]. In addition, it has been shown that this metric does not interfere with the quantification of binocular summation for this visual function [73]. The higher the contrast sensitivity value, the better the visual performance.

#### 2.4.2. Stereoacuity

Stereopsis was evaluated by means of near and distance stereoacuity under photopic conditions in such a way that the higher the stereoacuity value, the worse the stereopsis. Firstly, distance stereoacuity was assessed at 5.5 m using the differentiated stereo D8 polarized test of the Pola VistaVision monitor (DMD MedTech, Villarbasse, Torino, Italy). To evaluate distance stereoacuity, polarized vertical lines were displayed on the monitor [30]. For each disparity, the participant, wearing polarized glasses, had to recognize which of the five vertical lines displayed on the monitor was perceived stereoscopically (crossed disparity). A total of eight disparities (from 300 to 10 s of arc) were evaluated. Secondly, stereoacuity at a near distance was measured using the Frisby stereo test (Stereotest Ltd., Sheffield, UK), which represents a reliable test to evaluate stereopsis [74]. The test consists of three plates of different thicknesses (6, 3, and 1.5 mm). Each plate contains four squares of random patterns composed of arrowheads of various sizes. These patterns are printed on one side of the plate, but one of them has a central circular portion printed on the opposite side, which generates a certain disparity and depth perception. The task of the subject consists of recognizing the circle stereoscopically perceived for each plate and for different near distances. The Frisby stereotest allows us to evaluate a range of stereoacuities between 600 and 5 arcsecs depending on the thickness of the plate and the viewing distance. The smaller the thickness of the plate and the greater the viewing distance, the smaller the disparity induced and the lower the stereoacuity value, the better the stereopsis. Finally, an overall stereoacuity score (OSS) was calculated by averaging the z-scores of all stereoacuities at distance and near in all the experimental conditions (wearing filters and after alcohol intake). A z-score represents a measurement of how many standard deviations an individual value is away from the group mean and has been widely used in research [5,11,27,75]. For the OSS, the more positive the score, the higher the stereoacuity value and the worse the stereopsis.

#### 2.4.3. Visual Discrimination Capacity

We evaluated visual discrimination capacity under low illumination conditions using the halo test, which is based on the freeware software Halo v1.0 (University of Granada, Granada, Spain; http://hdl.handle.net/10481/5478, accessed on 14 December 2022). The test consists of detecting peripheral stimuli presented randomly around the central stimulus under low illumination conditions. The light stimuli were presented on a monitor located 2.5 m from the observer’s position. A 30-pixel radius was set for the central stimulus and a 1-pixel radius for the peripheral stimuli, subtending 0.46 and 0.02 degrees, respectively, from the observer’s position. Each peripheral stimulus was presented in one of the four possible positions per semi-axis out of a total of 15 semi-axes. This central higher-luminance stimulus is responsible for the perception of halos and other night vision disturbances, such as glare or starbursts. During the test, the participant had to press the right mouse button each time a peripheral luminous stimulus was detected. At the end of the test, a visual disturbance index (VDI) was obtained, quantifying the dysphotopsia. This index is defined by considering the undetected stimuli versus the total stimuli presented to the subject: the higher the VDI, the higher the halo size and the worse the visual discrimination capacity of the participant. This parameter is widely employed in clinical applications, such as ocular pathologies [53,57,76], refractive surgery [77], or monovision technique [26] to quantify night-vision disturbances.

### 2.5. Assessment of Ocular Parameters

#### 2.5.1. Retinal Image Quality

The OQAS II double-pass device (Optical Quality Analysis System II, Visiometrics, Terrassa, Spain) was used to assess retinal image quality. It has been widely used and validated in clinical practice [57,78,79]. We used three objective parameters for evaluating retinal image quality: the objective scatter index (OSI), the Strehl ratio (SR) and the MTF (Modulation Transfer Function) cutoff. The OSI quantifies the intraocular scattering in the outer part of the double-pass image for an artificial pupil size of 4 mm. It takes into account the light intensity within an annular area of 12 and 20 arc min (near-angle scattering) with respect to the central peak of the double-pass image: the higher the OSI value, the higher the amount of intraocular scattering affecting the retinal image quality. A normal value for this parameter is lower than 1.0 [80]. The second is the Strehl ratio which is defined as the ratio between the 2D-MTF area of the eye and the diffraction-limited 2D-MTF area for an artificial pupil size of 5 mm (in our study), ranging between 0 to 1 in such a way that the higher this value, the lower the ocular aberrations and intraocular scattering and, therefore, the better the retinal image quality. On the other hand, the MTF cutoff represents the spatial frequency corresponding to a theoretical MTF value of 0 (the noise produced by the CCD camera is considered in the calculation of the parameter) for an artificial pupil of 5 mm. The lower the MTF cutoff value, the worse the retinal-image quality. In each condition and parameter, three measurements were recorded and averaged.

#### 2.5.2. Straylight

The intraocular straylight was measured with the C-Quant device (Oculus GmbH, Wetzlar, Germany) which uses a compensation comparison method between two test field halves (randomly chosen). This device is extensively used in clinical practice [62,81,82]. In this visual test, the participant had look into the device to recognize which of the two semicircular fields was flickering the most. One of these two flickers depends on straylight and the other depends on the combination of straylight and compensation light [83]. At the end of the test, the ocular parameter log(s) (logarithm of the straylight) was obtained. This parameter is defined by the ratio between the scattered and non-scattered light so that the higher this parameter, the higher the intraocular straylight and, consequently, the higher the deterioration of the visual quality. This parameter depends on age, with a normal value of around 0.90 in young, healthy [84]. In each condition and monocularly (for both eyes), three measurements were performed to improve the accuracy of the test. Only measurements with a standard deviation of less than 0.08 were taken into account.

### 2.6. Interocular Differences

Interocular differences (ID) were evaluated by the variation between the non-dominant and dominant eye of the subject. We calculated the ID (in absolute values) for all the retinal image quality parameters (OSI, MTF cutoff, and SR) [27,34,85] and straylight (log(s)). In this study, we determined the ID under natural conditions for both eyes, but also between the dominant eye wearing the corresponding filter (BPM2 or BF_0.8) and the non-dominant eye under natural conditions (no filter). In addition, the IDs were calculated by wearing the BF_0.8 foil simultaneously on both eyes. Taking into account these filter conditions, the IDs were evaluated under two experimental conditions: under normal conditions (baseline, without alcohol consumption) and after alcohol consumption (aAC). Similarly to the aforementioned OSS, an overall interocular difference score (OIDS) was obtained for each participant by averaging the z-scores of all the interocular differences for the ocular parameters studied (OSI, MTF cut-off, SR and log (s)) in the experimental conditions evaluated (baseline and aAC). For all the variables, the more positive the OIDS score was, the greater the interocular differences were.

### 2.7. Binocular Summation

We calculated the binocular summation ratio to characterize binocular vision. Binocular summation (*BS*) specifies the performance of binocular visual perception compared to the best monocular one in a determined psychophysical experiment [23,34]. In this study, *BS* was assessed for three visual functions: visual acuity (*VA*), contrast sensitivity (*CSF*), and visual discrimination capacity (*VDI*). Binocular summation was calculated according to the visual function. If the visual function was such that a high value of the function implies a better visual outcome (such as visual acuity or contrast sensitivity), then the binocular summation was calculated by dividing the value of the binocular visual function by the best monocular value of that function (the highest of the two monocular values), as shown in Equation (1) [27,34,86,87]:

(1) $$BS_{VF}=\frac{VF_{bin}}{VF_{best\_mon}}$$

where *VF* refers to a visual function (*VA* or *CSF*), and *BS_VF_*, *VF_bin_*, *VF_best_mon_* are the binocular summation for the visual function (*BS_VA_* or *BS_CSF_*); the binocular visual function (*VA_bin_* or *CSF_bin_*); and the best monocular visual function (*VA_best_mon_* or *CSF_best_mon_*), respectively. Otherwise, if the visual function was such that a high value of the visual parameter implies a worse visual outcome, as occurs with the *VDI* in visual discrimination capacity, the binocular summation for this function, *BS_VDI_*, was calculated as the inverse of Equation (1), thus, dividing the best monocular *VDI* value, *VDI_best_mon_* (i.e., the lowest *VDI*) by the binocular *VDI* (Equation (2)), as shown in other works [26].

(2) $$BS_{VDI}=\frac{VDI_{best\_mon}}{VDI_{bin}}$$

A *BS* value greater than 1 indicates a positive binocular summation (superiority of the binocular system compared to best monocular eye). In the opposite, a *BS* value lower than 1 refers to the inhibition of the binocular system and consequently, the superiority of the best monocular eye with respect to the binocular one. We calculated this ratio for each filter condition under the two experimental conditions analyzed (baseline and after alcohol consumption). In the same way, as it was performed for stereopsis or interocular differences, an overall binocular summation score (OBSS) was also calculated, taking into consideration the binocular summation for the visual functions analyzed (*VA*, *CSF*, and *VDI*). We averaged the z-scores of these visual variables for each filter and for each alcohol consumption condition. The lower the OBSS, the lower the binocular summation and the poorer the binocular visual performance.

### 2.8. Statistical Analysis

For the data analysis, we used the SPSS 26.0 software package (SPSS Inc., Chicago, IL, USA). We analyzed the normal distribution of all the parameters (Shapiro-Wilk test). In the case of normal distribution, a t-test for two-sided alternatives was performed to compare each visual variable used (with the same filter condition) between the two experimental conditions (baseline and aAC). Similarly, the Wilcoxon signed-rank test was used in the case of no normal distribution. Comparing the different filter conditions under the same experimental condition (baseline and after alcohol consumption), a Friedman test using a two-way ANOVA was conducted with Bonferroni correction. Finally, Spearman’s rank correlation coefficient (or Spearman’s ρ) was calculated between the overall interocular difference score of the ocular parameters (OIDS) and the overall binocular summation score of the visual functions (OBSS), and between the overall interocular difference score of the ocular parameters (OIDS) and the overall stereoacuity score of the visual functions (OBSS). A statistical significance level of 95% was applied for all tests (*p* < 0.05).

## 3. Results

### 3.1. Monocular Visual Function

Table 1 shows the monocular mean values of VA, CSF and VDI under different filter conditions (no filter, BPM2 fog filter and Bangerter foil of 0.8) for the two experimental sessions: baseline (without alcohol consumption) and aAC (after alcohol consumption, with a BrAC of 0.40 mg/L). After alcohol consumption, significant impairments were found in the monocular visual functions studied for all filter conditions (VA: t(25) = 2.703, *p* = 0.012; CSF: t(25) = 2.944, *p* = 0.007; VDI: Z = −3.377, DF = 25, *p* = 0.001). In the baseline condition, we found significant impairment in all monocular visual functions studied for the dominant eye wearing the Bangerter foil (DE with BF_0.8): VA (χ^2^(2) = 1.346, *p* < 0.001), CSF (χ^2^(2) = 1.327, *p* < 0.001) and VDI (χ^2^(2) = −1.173, *p* < 0.001) except for BPM2 condition for VA (χ^2^(3) = 0.615, *p* = 0.514) and CSF (χ^2^(3) = 0.462, *p* = 0.999). For the baseline and after-alcohol consumption conditions, no statistically significant differences were found in the no-filter condition between the dominant eye (DE) and the non-dominant eye (NDE) in all monocular visual functions (*p* > 0.05). Similarly, after alcohol consumption, all monocular visual functions for the BF_0.8 condition (DE wearing the BF_0.8) were significantly impaired with respect to no filter condition (*p* < 0.001), except for the BPM2 condition for VDI (χ^2^(3) = 0.731, *p* = 0.248) and CSF (χ^2^(3) = 0.500, *p* = 0.976) Therefore, monocular visual performance was negatively affected after alcohol consumption (BrAC = 0.40 mg/L) and simulated visual deterioration (BPM2 fog filter and Bangerter foil of 0.8).

### 3.2. Binocular Visual Performance

**Figure 1:**
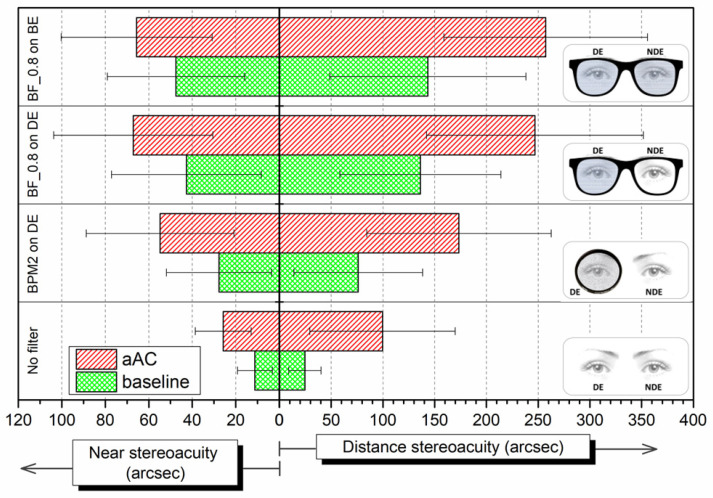
Bar graph of mean stereoacuity (near and distance) values in baseline and after alcohol consumption (aAC) for the different filter conditions: no filter; BMP2 fog filter on the dominant eye (BMP2 on DE); Bangerter foil of 0.8 on the dominant eye (BF_0.8 on DE); and Bangerter foil of 0.8 on both eyes (BF_0.8 on BE). Standard deviations included. NDE: non dominant eye.

Table 2 shows the mean binocular values of VA, CSF, and VDI in the baseline condition and after alcohol consumption (BrAC = 0.40 mg/L) for the different filter conditions: no filter, BPM2 fog filter on the dominant eye (BPM2 on DE), Bangerter foil BF_0.8 on the dominant eye (BF_0.8 on DE) and BF_0.8 foil on both eyes (BF_0.8 on BE). For all binocular visual functions, the results were significantly better for the baseline and no-filter conditions. For each filter condition, all binocular visual functions significantly deteriorated after consuming alcohol (BrAC = 0.40 mg/L) compared to the baseline session: VA (Z = −3.449, DF = 25, *p* = 0.001), CSF (t(25) = 2.535, *p* = 0.018), VDI (*p* < 0.001), distance stereoacuity (*p* < 0.001) and near stereoacuity (Z = −2.174, DF = 25, *p* = 0.030). Stereoacuity in distance vision was especially impaired after alcohol consumption for all filter conditions (Figure 1). In the baseline condition, significant deteriorations in VA, CSF and VDI were found for all filters compared to the natural condition (no filter): VA: χ^2^(3) = 1.288, *p* = 0.002; CSF: χ^2^(3) = 1.019, *p* = 0.027; VDI: χ^2^(3) = −1.019, *p* = 0.027. Comparing each condition to the worst result (BF_0.8 on BE), we found significant impairments for VA (χ^2^(3) = 1.327, *p* = 0.001), CSF (χ^2^(3) = 1.250, *p* = 0.003) and VDI (χ^2^(3) = −1.327, *p* = 0.001) in all conditions. After alcohol consumption, significant impairments in VA, CSF, and VDI were found for all filters compared to natural condition (VA: χ^2^(3) = 1.096, *p* = 0.013; CSF: χ^2^ (3) = 1.212, *p* = 0.004; VDI: χ^2^(3) = −1.327, *p* = 0.001), except for BPM2 condition for VA (χ^2^(3) = 0.923, *p* = 0.060) and VDI (χ^2^(3) = −0.769, *p* = 0.190). With respect to the worst result (BF_0.8 on BE), significant worsening was observed when this condition was compared with all of the filters (VA: χ^2^(3) = 1.346, *p* = 0.001; CSF: χ^2^(3) = 1.404, *p* = 0.001; VDI: χ^2^(3) = −1.269, *p* = 0.002).

Regarding stereopsis, Figure 1 shows the mean values and standard deviations for near and distance stereoacuity under the different experimental conditions (filter and alcohol consumption). For near and distance stereoacuity, in the baseline condition and after alcohol consumption (aAC), significant increases were found in all filters as compared to no filter (At near, baseline: χ^2^(3) = −1.115, *p* = 0.011; aAC: χ^2^(3) = −1.346, *p* = 0.001; At a distance, baseline: χ^2^(3) = −1.135, *p* = 0.009; aAC: χ^2^(3) = −1.077, *p* = 0.016). In addition, for distance stereoacuity, significant deteriorations were found for BF_0.8 condition (BF_0.8 on DE and BF_0.8 on BE) compared to the BPM2 condition (baseline and aAC: χ^2^(3) = −0.981, *p* = 0.037). In summary, binocular visual performance significantly deteriorated following alcohol intake (BrAC = 0.40 mg/L) for all visual functions, but also for wearing filters, both in the baseline condition and under the influence of alcohol (the worst situation bring for BF_0.8 on BE).

Table 3 shows the mean interocular differences for retinal image quality parameters (SR, MTF cut-off and OSI) and for straylight (log(s)) in the two experimental conditions: baseline and after alcohol intake (BrAC = 0.40 mg/L). Mainly, for each filter condition, no significant changes in interocular differences were found after alcohol consumption compared to the baseline condition in all ocular parameters measured, except for: BF_0.8 on DE in MTF cut-off (t(25) = 2.789, *p* = 0.010) and SR (t(25) = 3.670, *p* = 0.001), BF_0.8 on BE in log(s) (Z = −2.974, DF = 25, *p* = 0.003) and SR (Z = −2.046, DF = 25, *p* = 0.041), and BPM2 on DE in OSI (Z = −2.740, DF = 25, *p* = 0.006). For the straylight log(s), we found a significant increase in interocular differences in the baseline condition (no alcohol consumption) when comparing natural viewing (no filter) with all of the filter conditions (χ^2^(3) = −1.404, *p* = 0.001). Similarly, after alcohol consumption, the interocular differences significantly increased (χ^2^(3) = −1.596, *p* < 0.001), excluding BF_0.8 on BE condition (*p* > 0.05). For the OSI, in the baseline condition and after alcohol consumption (aAC), the interocular differences were significantly larger for the BF_0.8-on-DE condition compared to the rest of the filter conditions (Baseline: χ^2^(3) = −1.327, *p* = 0.001; aAC: χ^2^(3) = −1.000, *p* = 0.031). A significant increase in interocular differences was also found comparing natural viewing with BF_0.8 on BE (Baseline: χ^2^(3) = −1.058, *p* = 0.019; aAC: χ^2^(3) = −1.538, *p* < 0.001). For SR and MTF cut-off, in the baseline condition and after alcohol consumption, the interocular differences wearing BF_0.8 foils on both eyes were significantly lower compared to the other filter conditions (Baseline, SR: χ^2^(3) = 1.442, *p* < 0.001 and MTF: χ^2^(3) = −1.115, *p* = 0.011; aAC, SR: χ^2^(3) = 0.962, *p* = 0.043 and MTF: χ^2^(3) = 1.000, *p* = 0.031). In addition, the interocular differences were significantly larger wearing the BF_0.8 on the dominant eye compared to the rest of the filter conditions (Baseline, SR: χ^2^(3) = −0.962, *p* = 0.043 and MTF: χ^2^(3) = 1.115, *p* = 0.011; aAC, SR: χ^2^(3) = −1.000, *p* = 0.031 and MTF: χ^2^(3) = −1.308, *p* = 0.002).

Table 4 shows mean binocular summations for all visual parameters (VA, CSF and VDI) in baseline and after alcohol consumption (BrAC = 0.40 mg/L) and for all filter conditions. Under these conditions, all binocular summations were higher than 1, proving the superiority of the binocular system compared to the monocular one for all filters, except for the binocular summation of VA, CSF (in the baseline condition) and VDI (after alcohol consumption) for BF_0.8 on DE.

Considering each of the filter conditions, no significant differences were found in binocular summations for VA and VDI between baseline and aAC conditions. However, the binocular summation of the CSF significantly increased after alcohol consumption with respect to the baseline for all filters (no filter: Z = −2.693, DF = 25, *p* = 0.007; BF_0.8 on DE: Z = −2.566, DF = 25, *p* = 0.010; BF_0.8 on BE: t(25) = −2.123, *p* = 0.044) except for BPM2 (Z = −1.448, DF = 25, *p* = 0.148).

In the baseline condition, the highest binocular summation of the VA was obtained with BF_0.8 on BE (1.19 ± 0.19), being significantly higher than for the other filters (*p* < 0.001) except for no filter (χ^2^(3) = −0.558, *p* = 0.716). The lowest binocular summation of the VA (0.96 ± 0.11) was found for BF_0.8 on DE and was significantly lower than for the other filters (χ^2^(3) = 1.404, *p* = 0.001) except for the BPM2 (*p* > 0.05). After alcohol consumption, no significant differences were observed for binocular summation of the VA, comparing all filters with each other (*p* > 0.05) except for the two conditions wearing the BF_0.8 (χ^2^(3) = −1.038, *p* = 0.022). For the CSF, in the baseline condition, the binocular summation wearing the BF_0.8 on both eyes was also significantly higher than binocular summation for the other filters (χ^2^(3) = −1.077, *p* = 0.016) except for the natural condition (*p* > 0.05). The binocular summation wearing the BF_0.8 on DE was significantly lower than binocular summation for natural conditions (χ^2^(3) = 1.346, *p* = 0.001). After alcohol consumption, the binocular summation of BF_0.8 on BE was significantly higher than the binocular summation of BF_0.8 on DE and BPM2 (χ^2^(3) = −0.962, *p* = 0.043). For the VDI, in baseline and after alcohol consumption, the binocular summation of BF_0.8 on DE was significantly lower than natural and wearing the BF_0.8 on both eyes’ conditions (Baseline: χ^2^(3) = −1.154, *p* = 0.008; aAC: χ^2^(3) = −1.077, *p* = 0.016). Therefore, binocular summation for the three visual parameters (VA, CSF and VDI) was negatively affected by the filter (except for BF_0.8 on BE) but less after alcohol consumption.

**Figure 2:**
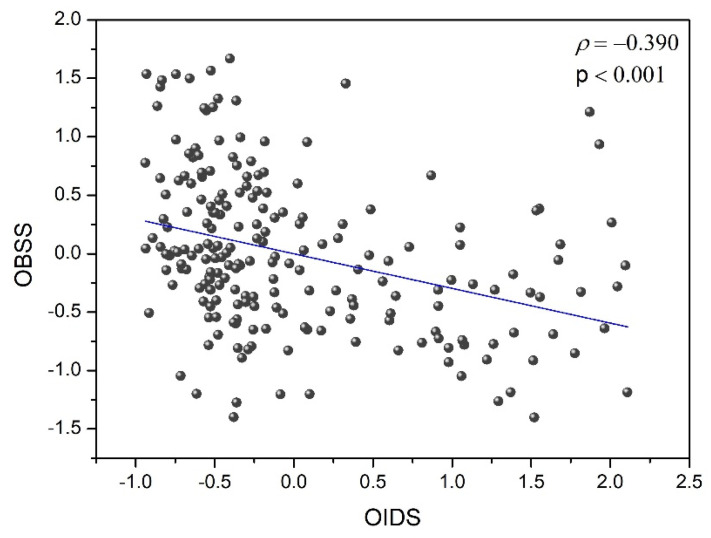
The overall binocular summation score (OBSS) of the visual function as a function of the overall interocular difference s/core (OIDS) under the two experimental conditions (baseline and after alcohol intake).

Figure 2 shows the relationship between the overall binocular summation score (OBSS) for the visual function and the overall interocular difference score (OIDS) of the ocular parameters. A significant negative correlation was found between OBSS and OIDS (ρ = −0.390, *p* < 0.001) considering scores obtained with all filters and under the two experimental conditions (baseline and aAC). Despite the spread of data, this significant correlation revealed that the higher the interocular differences for ocular parameters analyzed, the lower the binocular summation for the visual functions measured (baseline and after alcohol consumption). As a result, an improved binocular visual performance was in relation to a decrease in interocular differences.

**Figure 3:**
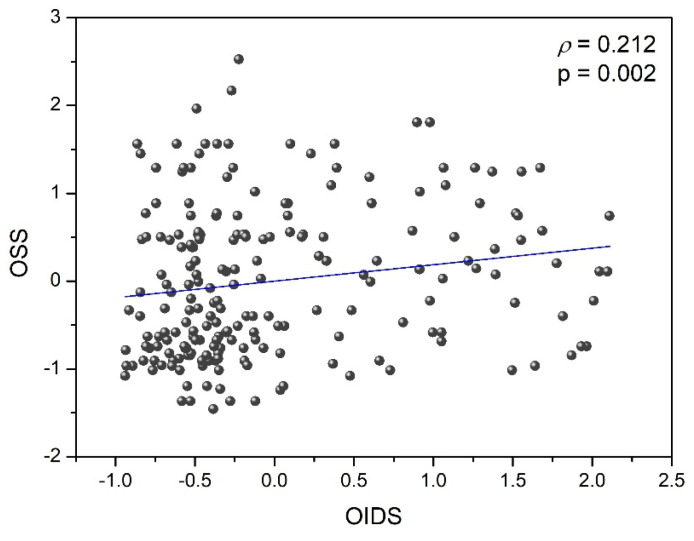
The overall stereoacuity score (OSS) as a function of the overall interocular difference score (OIDS) under the two experimental conditions (baseline and after alcohol intake).

Figure 3 shows the relationship between the overall stereoacuity score (OSS) and the overall interocular difference score (OIDS) of the ocular parameters studied in the baseline and after alcohol consumption. A significant positive correlation was obtained between OSS and OIDS (ρ = 0.212, *p* = 0.002) in such a way that the higher the interocular differences for ocular parameters analyzed, the higher the stereoacuity measured in this study (combining distance and near stereoacuity) and consequently, the worse the stereopsis and the binocular visual performance. As a result, taking into consideration both correlations (Figure 2 and Figure 3), binocular visual performance (binocular summation and stereopsis) was negatively influenced by interocular differences of ocular parameters in the experimental conditions studied (baseline and aAC). 

## 4. Discussion

This study allowed further knowledge on the performance of binocular vision after inducing visual deterioration through different experimental conditions, including alcohol consumption. Firstly, monocularly, we found significant visual deterioration in visual acuity, contrast sensitivity, and visual discrimination capacity (by means of VDI) due to moderate alcohol consumption (mean BrAC of 0.40 mg/L). Watten and Lie [3] showed that visual acuity and contrast sensitivity deteriorated under alcohol consumption (Blood Alcohol Concentration, mean BAC = 0.10% equivalent to a mean BrAC of 0.50 mg/L). Similarly, Casares-Lopez et al. [88] found a significant deterioration, in contrast, sensitivity, visual acuity and visual discrimination capacity (VDI) under a mean alcohol content of BrAC = 0.34 mg/L. Castro et al. [9] also reported significant monocular deterioration in VDI through a comparable alcohol content (mean BrAC = 0.32 mg/L). Following the aforementioned studies, our study is in accordance with the deteriorations found by them. However, it should be noted that in the studies of Casares-Lopez et al. and Castro et al., the participants reached different BrACs and the results refer to the average BrAC for all participants. In our study, a constant BrAC of 0.40 mg/L was maintained for all participants, so the results indicate the effect of that BrAC on the visual performance of different participants. This is an important aspect since this BrAC corresponds to the legal limit for driving in forty-five countries around the world, including the United Kingdom and the United States of America [1]. In a previous work, we evaluated the influence of moderate alcohol consumption (0.40 mg/L) on binocular vision and driving performance [11]. However, we evaluated binocular vision by means of visual acuity and stereopsis in far vision but also the vergence system through phorias and fusional vergences, but without evaluating the effect of possible interocular differences [11]. In the present study, we evaluated how interocular differences (natural and filter-induced) may affect other relevant aspects of binocular vision, including binocular summation for different visual functions, under natural conditions but also for moderate alcohol consumption. Under simulated visual deterioration Induced by Bangerter foil of 0.8 (BF_0.8) and BPM2 fog filter, a significant impairment was observed in visual acuity with respect to no filter condition according to other studies, such as that of Williamson et al. [89] who also found a similar expected degree of visual deterioration (0.8 in decimal). Odell et al. [90] also found a visual acuity impairment with the use of this filter. Still, they did not reveal a change in contrast sensitivity, contrary to our study, which highlighted significant deterioration with Bangerter foil of 0.8 (BF_0.8) and BPM2 fog filter in this visual parameter, also corroborated by other studies [27,69]. For visual discrimination capacity, our results confirmed the findings of Castro et al. [69], which found monocular deterioration induced by a wide range of Bangerter foils, including 0.8 and fog filters, but we also demonstrated a greater impairment of this visual function wearing the BF_0.8 and BPM2 after alcohol consumption.

Secondly, for binocular vision, contrast sensitivity also deteriorated under moderate alcohol consumption, as other authors have reported, such as Nicholson et al. [7] who found an impairment in the static and dynamic contrast sensitivity with a moderate breath alcohol concentration (BAC) of 0.043% (equivalent to a mean BrAC of 0.22 mg/L). Pearson et al. [4] suggested changes in contrast sensitivity specially at low and high spatial frequencies after a moderate alcohol consumption (mean BAC = 0.06% equivalent to a mean BrAC of 0.30 mg/L) which were confirmed by Casares-Lopez et al. with a BrAC of 0.32 mg/L [5]. In addition, in our study, the visual discrimination capacity, by means of the VDI, was also negatively impacted by a moderate alcohol consumption in accordance with Castro et al. [9] which found that the higher the breath-alcohol content (mean BrAC of 0.32 mg/L), the greater the deterioration of the visual-discrimination capacity using the same halo test. Our study has the strength that the visual results found are referenced to a constant BrAC of 0.40 mg/L, for all participants, which is also of special interest since it is the legal limit for driving in many countries, as previously mentioned. Likewise, stereopsis (evaluated by means of near and distance stereoacuity) negatively changed after alcohol consumption in line with Watten and Lie [3] where stereoacuity at near was affected for a BAC of 0.05% (mean BrAC of 0.25 mg/L) and 0.10% (mean BrAC of 0.50 mg/L). Ortiz-Peregrina et al. [14] also showed significant deterioration for stereoacuity far after alcohol intake (mean BrAC of 0.33 mg/L). It should be noted that stereopsis is an important aspect of the performance of daily tasks. In this sense, O’Connor et al. [18] proved that performance on motor skills tasks was related to stereoacuity, such as a worsening in near stereopsis negatively affecting these fine motor skills tasks (Pegboard test and bead threading). In addition, in another of their studies, O’Connor et al. [19] showed the relation between good sensory and motor fusion and an improvement in fine motor skills. In these studies, stereopsis was independently evaluated in near or far vision. However, in our work, we have demonstrated an impairment of stereopsis for both distances, being the strongest impairment for distance stereopsis. Regarding induced visual impairment, all binocular visual functions (visual acuity, contrast sensitivity, discrimination capacity and stereopsis) were negatively influenced by the three filter conditions (BPM2 on the dominant eye and BF_0.8 on the dominant and both eyes), with the greatest impairment being obtained with the BF_0.8 foil on both eyes and the least impairment with the BPM2 fog filter on the dominant eye. This was in accordance with other studies [27,42,91] corroborating the negative influence of intraocular scattering induced by filters on binocular vision. Although, as far as we know, our study is the first to consider the combined effects of alcohol consumption and penalizing filters (BPM2 and BF_0.8) on the binocular vision alteration.

Thirdly, binocular summation for VA, CSF and VDI decreased with a filter placed on the dominant eye (BPM2 and BF_0.8) but not with Bangerter foil of 0.8 on both eyes compared to baseline. This result indicates that increasing interocular differences deteriorates binocular summation for various visual functions, while if interocular differences are maintained, binocular summation does not change significantly. In this regard, according to Pardhan and Gilchrist [92], binocular summation for contrast sensitivity decreased with different levels of monocular defocus induced (from +0.50 to +3.50 D), i.e., increasing interocular differences. Furthermore, our results corroborated a previous study [27]: the higher the monocular visual degradation (on the dominant eye), the lower the binocular summation for these three visual parameters. In addition, in the present study, we have also studied that the increase of interocular differences deteriorated binocular summation for the aforementioned visual parameters with the effect of a moderate alcohol intake (BrAC of 0.40 mg/L). However, no significant differences were observed between baseline (no alcohol) and after alcohol consumption on binocular summation for VA and VDI, contrary to binocular summation for CSF being higher with moderate alcohol intake. This could be explained by higher monocular degradation—with respect to the binocular one—due to alcohol intoxication, in such a way that these higher ratios for contrast sensitivity after alcohol consumption could be misunderstood. This affirmation is in accordance with other studies. In their study, Casares et al. [5] also proved a greater monocular impairment on contrast sensitivity using the same CSF test as for the binocular ones for two different alcohol intakes (BrACs of 0.18 and 0.30 mg/L). Furthermore, another explanation could be considered on the fact that alcohol does not affect the contrast gain mechanism. Indeed, Pearson and Timney [93] showed that alcohol consumption (mean BAC of 0.06%, equivalent to a mean BrAC of 0.30 mg/L) impaired contrast discrimination performance, but it did not affect contrast gain mechanisms (important mechanisms underlying the visual system’s adaptation to the contrast of luminance in varying visual environments and related to binocular summation). On the other hand, as demonstrated in this study, interocular differences for ocular parameters were significantly correlated with binocular summation for visual functions, including contrast sensitivity, under both experimental conditions (filters and alcohol intake) such that the lower the interocular differences, the higher the binocular summation. Taking into account this consideration, our study showed that for some interocular differences for ocular parameters (Strehl ratio and MTF cut-off), the lowest values were found for the filter condition of BF_0.8 on both eyes. In addition, regarding changes in the interocular differences for ocular parameters after alcohol consumption, a moderate alcohol intake did not influence the number of interocular differences for the ocular parameters analyzed: OSI, MTF cut-off and Strehl ratio, obtained from the double-pass device OQAS; and straylight, log(s), provided by the C-Quant device. However, the interocular differences for log(s) were significantly lower for natural conditions (no filter) compared to the other filter conditions, in contrast to the three ocular parameters where no significant changes in interocular differences were noticed for two filter conditions (BPM2 on DE and BF_0.8 on BE). As explained in our previous reports [27,69], several differences are present between these two devices. Indeed, one (OQAS) provides objective measures, and the other one (C-Quant) uses a psychophysical method (compensation comparison method). In addition, for the calculation of the OSI parameter, the OQAS analyzes a light intensity of an annular area of 12 and 20 arc min of the double-pass image with respect to the intensity on the central peak (1 arc min). In contrast, for the Strehl ratio and MTF-cut off, this device analyzes an angular distribution lower than one degree. In contrast, the retinal straylight measured with the C-Quant device is calculated for a visual angle of 5 to 10 degrees. Furthermore, the OQAS uses near-infrared light (λ = 780 nm) for the measures, contrary to the C-Quant, which operates with a white light source.

Finally, in the present study, we highlighted a negative correlation (r = −0.390) between binocular summation for three main visual functions (VA, CSF and VDI) and interocular differences in ocular parameters (intraocular scattering, retinal-image quality by means of SR and MTF-cutoff, and straylight) considering all filters and alcohol consumption conditions: the higher the interocular differences for ocular parameters, the lower the binocular summation for visual function. This is in agreement with other studies that studied the influence of interocular differences (in optical quality parameters) on binocular visual performance through young, healthy subjects or amblyopia patients [27,60,89] but, in contrast to the present study, they did not investigate such differences after alcohol consumption. In fact, as aforementioned, moderate alcohol consumption did not impact interocular differences. This could explain why there should be no variation in binocular summation for VA and VDI between baseline condition and after alcohol intake. Additionally, the amount of impairment caused by alcohol consumption affected both eyes equally, so the interocular differences were hardly altered by alcohol intake.

Similarly, under these two experimental conditions (simulated visual deterioration induced by filters and alcohol consumption), a significant positive correlation (ρ = 0.212) was revealed between distance and near stereoacuity and overall interocular differences in ocular parameters in such a way that the higher the interocular differences in induced intraocular scattering, the greater the deterioration in stereopsis. This confirmed the findings of Zhao et al. [42], who measured the stereoacuity under different levels of scatter filter in three young, healthy subjects inducing various ranges of interocular differences, and found a deterioration of stereopsis due to an increase of interocular differences in this parameter.

In summary, in this present study, monocular and binocular vision through different visual functions measured, such as visual acuity, contrast sensitivity, visual discrimination capacity and distance and near stereoacuity were negatively affected by a moderate alcohol consumption (BrAC of 0.40 mg/L), but also by a simulated visual deterioration using filters. Due to the increment of intraocular scattering induced monocularly by the BPM2 fog filter (simulating an early cataract) and Bangerter foil of 0.8 on DE, the interocular differences for the ocular parameters (OSI, MTF cut-off, Strehl ratio and log(s)) significantly increased. However, interocular differences did not vary after alcohol consumption compared to the baseline condition. Similarly, the binocular visual performance through the calculation of binocular summation (VA, CSF and VDI) was proportionally degraded in the following order: natural condition (no filter), BPM2 on the dominant eye and Bangerter foil of 0.8 on the dominant eye, but not for Bangerter foil of 0.8 on both eyes. Binocular summation did not differ under alcohol consumption except for contrast sensitivity, being higher after alcohol intake for each filter condition measured. This could be interpreted by a higher monocular visual deterioration for this parameter with respect to the binocular one and a steady value in interocular differences. In fact, interocular differences and binocular vision were significantly correlated under both experimental conditions (filters and a moderate alcohol intake) in such a way that the higher the interocular differences, the lower the binocular summation and the higher the stereoacuity and, consequently, the worse the overall binocular visual performance. So, the combined effect of moderate alcohol consumption (BrAC of 0.40 mg/L) and simulated visual deterioration strongly impaired monocular and binocular visual function. Further investigation could be of interest to assess the influence of these experimental conditions (filter and alcohol) on a complex daily visual task, such as driving, where binocular vision plays an important role [11,94].

## 5. Conclusions

Monocular and binocular visual function, assessed through visual acuity, contrast sensitivity and visual discrimination capacity, strongly deteriorated after inducing visual impairment with filters but also after moderate alcohol consumption (BrAC = 0.40 mg/L). Near and distance stereopsis were also negatively affected by the filters and alcohol intake. Binocular summations for the aforementioned visual parameters decreased with filters but not under moderate alcohol consumption, highlighting for this condition a stronger monocular visual performance impairment with respect to the binocular one for this condition. Similarly, interocular differences for different ocular parameters (assessing intraocular scattering, retinal-image quality and straylight) increased for the different levels of forward scattering induced by filters but no change under a moderate alcohol intake. Finally, including all the values of the two experimental conditions (filters and alcohol consumption), significant correlations were found. Firstly, between binocular summation for visual function and interocular differences for ocular parameters and, secondly, between stereoacuity (at near and distance) and the same interocular differences tested. These correlations prove that the higher the interocular differences, the lower the binocular summation and the higher the stereoacuity and, therefore, the worse the overall binocular visual performance.

Our findings could be useful to understand better how binocular visual performance is affected by interocular differences as it may occur in ocular pathologies that initially affect one eye, such as amblyopia, keratitis and cataract. Furthermore, by taking into account moderate alcohol intoxication (which represents the most consumed psychoactive substance in the world and a global health problem), these findings should be of interest in daily visual tasks, such as driving, manual dexterity or fine motor skills.

## Figures and Tables

**Table 1:** Mean values of the monocular (mon) visual functions (Visual Acuity in decimal notation, VA; averaged Contrast Sensitivity Function, CSF; and Visual Disturbance Index, VDI) under two experimental conditions, baseline (no alcohol consumption) and aAC (after alcohol consumption), and for different monocular conditions (dominant eye, DE; non-dominant eye, NDE, dominant eye wearing the BPM2 fog filter, DE with BPM2; and dominant eye wearing the Bangerter foil, DE with BF_0.8). Standard deviations included and statistical results (t/Z) and *p*-values are indicated.

Monocular Visual Function	Filter	Baseline	aAC	t/Z; *p*-Value
VA (mon)	DE (no filter)	1.2 ± 0.1	1.0 ± 0.1	Z(25) = −4.228; *p* < 0.001
	NDE (no filter)	1.2 ± 0.2	0.9 ± 0.1	Z(25) = −4.317; *p* < 0.001
	DE with BPM2	0.9 ± 0.2	0.8 ± 0.2	t(25) = 2.703; *p* = 0.012
	DE with BF_0.8	0.8 ± 0.1	0.6 ± 0.1	Z(25) = −4.362; *p* < 0.001
CSF (mon)	DE (no filter)	149 ± 15	126 ± 20	Z(25) = −4.460; *p* < 0.001
	NDE (no filter)	146 ± 16	117 ± 26	Z(25) = −4.408; *p* < 0.001
	DE with BPM2	105 ± 33	85 ± 30	t(25) = 2.996; *p* = 0.006
	DE with BF_0.8	93 ± 20	77 ± 26	t(25) = 2.944; *p* = 0.007
VDI (mon)	DE (no filter)	0.151 ± 0.059	0.273 ± 0.158	Z(25) = −3.943; *p* < 0.001
	NDE (no filter)	0.177 ± 0.071	0.310 ± 0.155	Z(25) = −4.057; *p* < 0.001
	DE with BPM2	0.271 ± 0.172	0.543 ± 0.242	Z(25) = −4.229; *p* < 0.001
	DE with BF_0.8	0.540 ± 0.173	0.745 ± 0.204	Z(25) = −3.377; *p* < 0.001

**Table 2:** Mean values of the binocular (bin) visual functions (Visual Acuity in decimal notation, VA; averaged Contrast Sensitivity Function, CSF; and Visual Disturbance index, VDI) under the different experimental conditions, baseline (no alcohol consumption) and aAC (after alcohol consumption) and for different filter conditions: no filter, BPM2 fog filter on dominant eye (BPM2 on DE), Bangerter foil BF_0.8 on dominant eye (BF_0.8 on DE) and BF_0.8 on both eyes (BF_0.8 on BE). Standard deviations included and statistical results (t/Z) and *p*-values are indicated.

Binocular Visual Function	Filter	Baseline	aAC	t/Z; *p*-Value
VA (bin)	No filter	1.4 ± 0.1	1.1 ± 0.1	Z(25) = −4.396; *p <* 0.001
	BPM2 on DE	1.2 ± 0.1	1.0 ± 0.2	Z(25) = −3.891; *p <* 0.001
	BF_0.8 on DE	1.1 ± 0.1	1.0 ± 0.2	Z(25) = −3.449; *p* = 0.001
	BF_0.8 on BE	0.9 ± 0.1	0.7 ± 0.2	Z(25) = −3.743; *p <* 0.001
CSF (bin)	No filter	162 ± 10	147 ± 16	Z(25) = −4.168; *p <* 0.001
	BPM2 on DE	150 ± 14	128 ± 25	Z(25) = −3.983; *p <* 0.001
	BF_0.8 on DE	145 ± 14	130 ± 24	t(25) = 3.305; *p* = 0.003
	BF_0.8 on BE	108 ± 16	97 ± 24	t(25) = 2.535; *p* = 0.018
VDI (bin)	No filter	0.112 ± 0.028	0.205 ± 0.098	Z(25) = −4.130; *p <* 0.001
	BPM2 on DE	0.153 ± 0.058	0.296 ± 0.165	Z(25) = −3.899; *p <* 0.001
	BF_0.8 on DE	0.194 ± 0.094	0.348 ± 0.156	Z(25) = −4.184; *p <* 0.001
	BF_0.8 on BE	0.440 ± 0.162	0.642 ± 0.232	t(25) = −4.494; *p <* 0.001

**Table 3:** Mean values of the interocular differences for the ocular parameters analyzed: objective scatter index (OSI), modulation transfer function cut-off (MTF cut-off), Strehl ratio (SR) and straylight (log(s)) under the different experimental conditions: baseline (no alcohol consumption) and aAC (after alcohol consumption) and for different filter conditions: no filter, BPM2 fog filter on the dominant eye (BPM2 on DE), Bangerter foil BF_0.8 on the dominant eye (BF_0.8 on DE) and BF_0.8 on both eyes (BF_0.8 on BE). Standard deviations are included and statistical results (t/Z) and *p*-values are indicated.

Interocular Differences	Filter	Baseline	aAC	t/Z; *p*-Value
OSI	No filter	0.22 ± 0.20	0.25 ± 0.21	Z(25) = −0.644; *p* = 0.519
	BPM2 on DE	0.30 ± 0.22	0.61 ± 0.49	Z(25) = −2.740; *p* = 0.006
	BF_0.8 on DE	3.33 ± 0.95	3.38 ± 1.26	t(25) = −0.196; *p* = 0.846
	BF_0.8 on BE	0.81 ± 0.64	1.76 ± 2.31	Z(25) = −1.892; *p* = 0.058
MTF cut-off (cpd)	No filter	9.03 ± 6.57	7.14 ± 6.46	Z(25) = −1.333; *p* = 0.182
	BPM2 on DE	6.63 ± 6.98	7.54 ± 7.34	Z(25) = −0.292; *p* = 0.77
	BF_0.8 on DE	27.16 ± 10.27	22.98 ± 9.54	t(25) = 2.789; *p* = 0.01
	BF_0.8 on BE	1.98 ± 1.64	2.70 ± 1.97	Z(25) = −1.486; *p* = 0.137
SR	No filter	0.05 ± 0.04	0.04 ± 0.03	Z(25) = −1.090; *p* = 0.276
	BPM2 on DE	0.05 ± 0.04	0.04 ± 0.04	Z(25) = −0.902; *p* = 0.367
	BF_0.8 on DE	0.14 ± 0.06	0.10 ± 0.04	t(25) = 3.670; *p* = 0.001
	BF_0.8 on BE	0.01 ± 0.01	0.01 ± 0.01	Z(25) = −2.046; *p* = 0.041
log(s)	No filter	0.08 ± 0.09	0.08 ± 0.08	Z(25) = −0.764; *p* = 0.445
	BPM2 on DE	0.26 ± 0.12	0.31 ± 0.21	Z(25) = −0.737; *p* = 0.461
	BF_0.8 on DE	0.28 ± 0.19	0.31 ± 0.13	t(25) = −0.858; *p* = 0.399
	BF_0.8 on BE	0.23 ± 0.10	0.12 ± 0.12	Z(25) = −2.974; *p* = 0.003

**Table 4:** Mean values of the binocular summations (Visual Acuity, VA; averaged Contrast Sensitivity Function, CSF; and Visual Disturbance index, VDI) under the different experimental conditions, baseline (no alcohol consumption) and aAC (after alcohol consumption) and for different filter conditions: no filter, BPM2 fog filter on the dominant eye (BPM2 on DE), Bangerter foil BF_0.8 on the dominant eye (BF_0.8 on DE) and BF_0.8 on both eyes (BF_0.8 on BE). Standard deviations included.

Experimental Condition	Filter	Binocular Summation		
		VA	CSF	VDI
Baseline	No filter	1.11 ± 0.08	1.10 ± 0.12	1.35 ± 0.41
	BPM2 on DE	1.00 ± 0.11	1.03 ± 0.10	1.21 ± 0.44
	BF_0.8 on DE	0.96 ± 0.11	1.00 ± 0.13	1.01 ± 0.43
	BF_0.8 on BE	1.19 ± 0.19	1.19 ± 0.19	1.31 ± 0.42
aAC	No filter	1.09 ± 0.12	1.19 ± 0.14	1.30 ± 0.32
	BPM2 on DE	1.06 ± 0.16	1.12 ± 0.22	1.13 ± 0.38
	BF_0.8 on DE	1.03 ± 0.16	1.15 ± 0.25	0.92 ± 0.28
	BF_0.8 on BE	1.17 ± 0.27	1.33 ± 0.31	1.25 ± 0.38

## Data Availability

Available from the corresponding author on reasonable request.
